# Grafts Derived from an *α*-Synuclein Triplication Patient Mediate Functional Recovery but Develop Disease-Associated Pathology in the 6-OHDA Model of Parkinson’s Disease

**DOI:** 10.3233/JPD-202366

**Published:** 2021-04-13

**Authors:** Shelby Shrigley, Fredrik Nilsson, Bengt Mattsson, Alessandro Fiorenzano, Janitha Mudannayake, Andreas Bruzelius, Daniella Rylander Ottosson, Anders Björklund, Deirdre B. Hoban, Malin Parmar

**Affiliations:** a Wallenberg Neuroscience Center, Department of Experimental Medical Science, Lund University, Lund, Sweden; b Lund Stem Cell Center, Lund University, Lund, Sweden

**Keywords:** Parkinson’s disease, alpha-synuclein, cell transplantation, dopaminergic neurons

## Abstract

**Background:**

Human induced pluripotent stem cells (hiPSCs) have been proposed as an alternative source for cell replacement therapy for Parkinson’s disease (PD) and they provide the option of using the patient’s own cells. A few studies have investigated transplantation of patient-derived dopaminergic (DA) neurons in preclinical models; however, little is known about the long-term integrity and function of grafts derived from patients with PD.

**Objective:**

To assess the viability and function of DA neuron grafts derived from a patient hiPSC line with an *α*-synuclein gene triplication (AST18), using a clinical grade human embryonic stem cell (hESC) line (RC17) as a reference control.

**Methods:**

Cells were differentiated into ventral mesencephalic (VM)-patterned DA progenitors using an established GMP protocol. The progenitors were then either terminally differentiated to mature DA neurons *in vitro* or transplanted into 6-hydroxydopamine (6-OHDA) lesioned rats and their survival, maturation, function, and propensity to develop *α*-synuclein related pathology, were assessed *in vivo*.

**Results:**

Both cell lines generated functional neurons with DA properties *in vitro*. AST18-derived VM progenitor cells survived transplantation and matured into neuron-rich grafts similar to the RC17 cells. After 24 weeks, both cell lines produced DA-rich grafts that mediated full functional recovery; however, pathological changes were only observed in grafts derived from the *α*-synuclein triplication patient line.

**Conclusion:**

This data shows proof-of-principle for survival and functional recovery with familial PD patient-derived cells in the 6-OHDA model of PD. However, signs of slowly developing pathology warrants further investigation before use of autologous grafts in patients.

## INTRODUCTION

Parkinson’s disease (PD) is one of the most common neurodegenerative disorders, affecting millions of people worldwide. It is characterized primarily by the loss of dopaminergic (DA) neurons in the substantia nigra *pars compacta*, degeneration of the nigrostriatal pathway, and the presence of pathological protein inclusions known as Lewy bodies. For decades cell replacement therapy, where the aim is to replace the lost DA neurons with new healthy ones, has been explored as a treatment for PD [1]. In early clinical trials, patients were transplanted with DA progenitors obtained from fetal ventral mesencephalic (VM) tissue [2, 3]. These efforts showed proof-of-principle for graft survival and long term clinical benefit, albeit with varying outcomes [4–8]. However, fetal tissue is scarcely available, and its use for transplantation is associated with a number of ethical and practical issues. Alternative cell sources are therefore required for wide use of cell replacement therapy in PD.

Recent advances in stem cell biology now make it possible to generate DA progenitors from human embryonic stem cells (hESCs) and human induced pluripotent stem cells (hiPSCs) *in vitro* with high similarity to authentic midbrain DA neurons [9–11]. These pluripotent stem cell-derived DA neurons represent a scalable source of cells that can be standardized, and quality controlled prior to transplantation. Additionally, the use of hiPSCs provides the option to use the patient’s own cells for transplantation. Autologous cell replacement therapy has the advantage that the patient may not require immunosuppressive drugs, therefore avoiding any associated complications [12–16].

In some patients who received fetal VM transplants, postmortem analysis revealed the presence of slowly developing pathological or phosphorylated *α*-synuclein (pSyn) inclusions within the grafted cells [17–21]. Currently, it remains unclear if patient-derived VM progenitors would be inherently more susceptible to develop pathology in the grafted DA neurons than cells from healthy donors over time. A number of *in vitro* studies suggest that patient-derived DA neurons, especially those from familial forms of PD, are more vulnerable to dysfunction and protein inclusions due to their disease-specific backgrounds [22–26]. However, only a small number of studies have investigated transplantation of patient-derived DA neurons in preclinical models of PD using hiPSCs derived from sporadic PD patients [11, 27–29]. None of these studies reported any evidence of pathology in the transplant, however, given the extensive time it takes for pathology to develop in patients who received fetal VM transplants (>10 years), it may be too early to observe any pathological changes in cells from sporadic PD patients in these models.

In this study, we used a hiPSC line derived from an individual with a triplication mutation in *SNCA,* the gene encoding for *α*-synuclein (*α*Syn). Mutations in the *SNCA* gene have been linked to familial forms of PD [30–32] and variation at this locus (4q22) is also the most significant risk factor for sporadic PD [33, 34]. The *α*Syn triplication mutation leads to three copies of the gene on the first allele and one copy on the second allele, totaling 4 copies of the *SNCA* gene. This results in a doubling of messenger RNA and *α*Syn protein expression [26, 31, 35]. Since *α*Syn forms a major component of Lewy body pathology [36], and the triplication mutation is associated with early onset and rapidly progressing PD [31], we hypothesized that *α*Syn pathology, if present, would develop earlier in these grafts. Therefore, to investigate how cells with *α*Syn triplication mature and function, and if they develop any pathology after extended time periods after transplantation, we transplanted DA neurons derived from the Alpha Synuclein Triplication line AST18 [35] into the 6-hydroxydopamine (6-OHDA) preclinical model of PD. RC17, a clinical grade hESC line that has been extensively used in a number of pre-clinical studies [10, 37–39] was grafted in parallel as a control.

## MATERIALS AND METHODS

### Terminal differentiation of DA neurons

hESCs (RC17; Roslin Cells) and hiPSCs (AST18) were differentiated into DA neurons as described in Nolbrant et al. 2017 [10]. Pluripotent cells were maintained on laminin-521 in iPS brew. Cells were passaged approximately every 7 days (or when reaching confluency) with EDTA using a seeding density of 2,500 cells/cm^2^ in iPS brew with Y-27632. For differentiation, on day 0 cells were plated on laminin-111 coated wells in differentiation medium (N2 medium + Y-27632 + SB431542 + Noggin + Shh-C24II + CHIR99021), they received a medium change on day 2, 4, and 7. On day 9 the medium was changed to N2 medium + FGF8b. Cells were replated on day 11 using replating medium (B27 medium + Y-27632 + BDNF + AA + FGF8b), they also received a medium change on day 14. On d16 cells were replated using terminal differentiation medium (B27 medium + BDNF + AA + GDNF + cAMP + DAPT), cells received a medium change every 2-3 days. From day 25 onwards, only 75 % of the medium was replaced to reduce the risk of cell detachment. Cells were fixed and analyzed after 35 days in culture.

### Electrophysiology

Electrophysiological recordings were performed on cells after 35 days in culture. Cells were cultured on coverslips and transferred to a recording chamber with constant flow of Krebs solution gassed with 95% O_2_ and 5% CO_2_ kept at room temperature (RT). The composition of the Krebs solution was (mM) 119 NaCl, 2.5 KCl, 1.3 MgSO4, 2.5 CaCl2, 25 Glucose and 26 NaHCO3. Multiclamp 700B (Molecular Devices) and pulled glass capillaries with a resistance of 3–7 MOhm filled with intracellular solution (mM) 122.5 potassium gluconate, 12.5 KCl, 0.2 EGTA, 10 Hepes, 2 MgATP, 0.3 Na3GTP and 8 NaCl adjusted to pH 7.3 with KOH were used for recordings. Data acquisition was performed with pClamp 10.2 (Molecular Devices); current was filtered at 0.1 kHz and digitized at 2 kHz. Cells with a neuronal morphology and clear from any surface debris were selected for recordings. Immediately after establishing whole-cell access resting membrane potential (RMP) was measured in current clamp mode, and cells were kept at a holding potential of –60 to –70 mV. 500 ms long current in rheobase injection steps from –20 to +  35pA at 5pA increments was performed for evoked action potentials. For measurements of inward sodium and delayed rectifying potassium currents cells were clamped at –70 mV and voltage-depolarizing steps were delivered for 100 ms at 10 mV increments. Data analysis was performed using Igor Pro 8.04 (Wavemetrics) with NeuroMatic package [40].

### Immunocytochemistry

Cells were fixed in 4% PFA for 15 min and washed twice using 0.1 M phosphate-buffered saline with potassium (KPBS, pH = 7.4). Before staining cells were washed once with KPBS and then incubated in blocking solution (KPBS containing 0.1% Triton-X and 5% serum specific to the species of the secondary antibody) for 1 h. Following this, the primary antibody in blocking solution was added overnight at 4C. The primary antibodies used were: rabbit anti-LMX1A (1:1000, Merck Millipore ab10533), mouse anti-FOXA2 (1:500, Santa Cruz Biotechnology sc101060), goat anti-OTX2 (1:2000, R&D Systems AF1979), rabbit anti-TH (1:1000, Merck Millipore ab152), mouse anti-TAU(HT7) (1:500, Thermo Fisher Scientific MN1000), chicken anti-MAP2 (1:10000, Abcam ab5392), goat anti-GIRK2 (1:200, Merck Millipore ab65096), and mouse anti-*α*-synuclein (1:250, BD Biosciences 610787). The next day cells were washed three times and incubated with fluorophore-conjugated secondary antibodies (1:200, Jackson ImmunoResearch Laboratories) and DAPI in blocking solution for 2 h at room temperature (RT). Cells were then washed with KPBS a further three times and stored at 4C until analysis.

### Animals

All procedures were performed in accordance with the European Union Directive (2010/63/EU) and approved by the local ethical committee at Lund University, as well as the Swedish Department of Agriculture (Jordbruksverket). Female Sprague-Dawley (SD) rats were purchased from Charles River Laboratories and female nude athymic rats (Hsd:RH-*Foxn*1^rnu^) were purchased from Envigo. All rats were housed in ventilated cages with *ad libitum* access to food and water under a 12- h light/dark cycle.

### In vivo experimental design

SD rats received a 6-OHDA medial forebrain bundle (MFB) lesion and the extent of the lesion was confirmed by amphetamine-induced rotation test after 4 weeks. Following this, the rats received cell transplantation surgery and were perfused 8 weeks later. SD rats received daily immunosuppression via intraperitoneal injection of cyclosporine (10 mg/ kg) to prevent graft rejection. Nude athymic rats received a 6-OHDA MFB lesion and the extent of the lesion was confirmed by the amphetamine-induced rotation test after 4 weeks. Following this, the rats received cell transplantation surgery and were perfused either 7 or 24 weeks later. Behavioural recovery was assessed by amphetamine-induced rotations at 16, 18, 20, 22, and 24 weeks post-transplantation.

### Preparation of DA progenitor cells for transplantation

hESCs (RC17; Roslin Cells) and hiPSCs (AST18) were differentiated into VM-patterned progenitor cells as described in Nolbrant et al. 2017 [10] and transplanted on day 16. All cell preparation batches passed quality control checks as in the protocol (Supplementary Figure 1).

### Surgeries

All surgeries were performed under general anesthesia using a solution of fentanyl (0.36 mg/kg) and medetomidine hydrochloride (dormitor; 0.36 mg/kg). Animals were placed into a stereotaxic frame and the tooth bar was adjusted to the flathead position. For the lesion surgery, 3*μ*L of 6-OHDA (3.5*μ*g/*μ*L of free base, dissolved in ascorbate-saline) was injected unilaterally into the MFB (AP –4.4, ML –1.1, DV –7.8) at a rate of 0.3*μ*L per minute. For the transplant surgery, 4*μ*L of cell suspension (75,000 c/*μ*L) was injected unilaterally at four sites in the striatum (SD rats: AP + 0.5, ML –3.0, DV –4.5/–5.5 and AP + 1.2, ML –2.6, DV –4.5/–5.5; nude athymic rats: AP + 0.9, ML –3.0, DV –4.0/–5.0 and AP+1.4, ML –2.6, DV –4.0/–5.0) at a rate of 1*μ*L per minute. After surgery, anesthesia was reversed with atipamezole (antisedan; 0.28 mg/kg) and analgesia was administered using buprenorphine (temgesic; 0.04 mg/kg).

### Behavioural testing

Rotational bias was assessed by amphetamine-induced rotations both before transplantation and at several timepoints after transplantation. Animals received an intraperitoneal injection of dexamphetamine solution (3.5 mg/kg) and were placed into automated rotometer bowls for 90 min (Omnitech Electronics Inc.). Full body turns towards the side of the lesion were given positive values and turns to the opposite side given negative values, with data expressed as net turns per minute. In the behavioural assessment, only animals with complete lesions (>4 net turns/min on the amphetamine rotation test at baseline) and confirmed TH^+^ cell loss in the substantia nigra *post-hoc* were included, resulting in *n* = 5 for the RC17 and *n* = 5 for the AST18 group.

### Immunohistochemistry

Rats were given terminal anesthesia with a lethal dose of sodium pentobarbitone injected intraperitoneally. Animals were transcardially perfused with physiological saline solution followed by ice-cold 4% PFA. Brains were post-fixed for 24 h in 4% PFA, transferred to 25% sucrose for 48 h and then sectioned coronally using a freezing microtome at a thickness of 35*μ*m (1:8 series). Immunohistochemistry wasperformed on free floating sections and all washing steps used 0.1 M phosphate-buffered saline with potassium (KPBS, pH = 7.4).

For DAB staining, sections were washed three times and then incubated in a quench solution for 15 min at RT. After washing a further three times, the sections were incubated in blocking solution (KPBS containing 0.25% Triton-X and 5% serum specific to the species of the secondary antibody) for 1 h. Following this, the primary antibody in blocking solution was added overnight at RT. The primary antibodies used were mouse anti-hNCAM (1:1000, Santa Cruz Biotechnology sc106) and rabbit anti-TH (1:2000, Merck Millipore ab152). The next day sections were washed twice and incubated in blocking solution for 30 min. The sections were incubated with secondary biotinylated antibodies (1:200, Vector Laboratories) for 1 h at RT. After washing a further three times, sections were incubated with avidin-biotin complex (ABC) for 1 h at RT for amplification. Next, sections were incubated in 0.05% DAB for 1–2 min before addition of 0.01% H_2_O_2_ for 1–2 min. After development, sections were mounted on gelatin-coated slides and then dehydrated in an ascending series of alcohols, cleared in xylene, and coverslipped with DPX mountant.

For fluorescent immunolabeling, sections were washed three times and then incubated in Tris-EDTA (pH 9.0) for 30 min at 80°C for antigen retrieval. After washing a further three times, the sections were incubated in blocking solution for 1 h. Following this, the primary antibody in blocking solution was added overnight at RT. The primary antibodies used were: rabbit anti-TH (1:2000, Merck Millipore ab152), sheep anti-TH (1:1000, Merck Millipore ab1542), mouse anti-*α*-synuclein (211) (1:2000, Santa Cruz sc12767), rabbit anti-IBA1 (1:1000 WAKO 019-19741), mouse anti-p-synuclein (81A) (1:10000, gift from Kelvin Luk University of Pennsylvania). The next day sections were washed twice and incubated in blocking solution for 30 min. The sections were incubated with fluorophore-conjugated secondary antibodies (1:200, Jackson ImmunoResearch Laboratories) for 1 h at RT. After washing a further three times, sections were mounted on gelatin-coated slides and coverslipped with PVA-DABCO containing DAPI (1:1000).

### Graft quantifications

Photomicrographs of hNCAM stained coronal sections were taken at the level of the striatum. To determine graft volume, the area of the graft core in every eighth section through the graft was measured using ImageJ (version: 2.0.0-rc-69/1.52p) and calibrated by associating the number of pixels with a known measurement, obtained from a scale taken as a photomicrograph using the same resolution and settings. The graft volume was calculated according to Cavalieri’s principle, given the known distance between each section and the known section thickness. To determine the DA neuron yield, the number of DAB-stained TH^+^ neurons in each section was counted manually using the Olympus AX70 inverted microscope at 20x magnification in brightfield. Final counts were adjusted for the number of series (1:8), and Abercrombie’s formula was used for correction of cell counts in histological sections to get an estimate of the total number of TH^+^ cells within the graft.

For the counts of pSyn^+^ pathology inside TH^+^ and IBA1^+^ cells, fluorescent images were taken on a TCS SP8 laser scanning confocal microscope at 20X objective magnification, and collected in a 3D stack (775×775*μ*m x *ca*25*μ*m). The scan resolution was 2048x2048 and scanning speed 200 MHz interval of every 1*μ*m (z-stack). Microglia size was calculated by determining the total volume of stained IBA1^+^ cells determined by threshold in the collected 3D stack, divided by the number of IBA1^+^ cells in each stack. Microglia density was calculated by the number of microglia/stack. TH^+^ and IBA1^+^ cells were identified and assessed for co-expression of pSyn^+^ inclusions using Volocity software. 15 animals were quantified (7 in the RC17 group and 8 in the AST18 group), with 5730 IBA1^+^ cells counted in total (mean 382 IBA1^+^ cells per animal) and 2703 TH^+^ cells counted in total (mean 180 TH^+^ cells per animal).

## RESULTS

### *α*Syn triplication does not significantly affect differentiation towards DA neurons in vitro

**Fig. 1:**
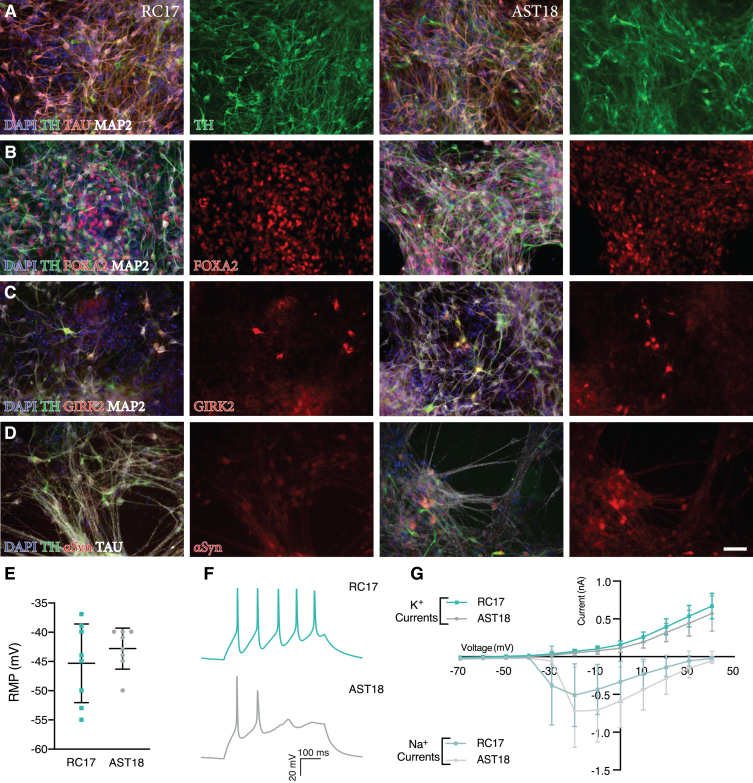
Analysis of terminally differentiated DA neurons at day 35 *in vitro*. Immunostaining of VM-patterned neurons showing (A) high expression of neuronal markers TAU and MAP2, and DA marker TH. Immunostaining showing co-expression of (B) TH, FOXA2, and MAP2, (C) TH, GIRK2, and MAP2, and (D) TH, *α*Syn, and TAU. Scale bar 50*μ*m. (E) Resting membrane potential (RMP) at day 35 measured by whole-cell patch-clamp recordings showing no major difference between the cell lines, *n* = 8 in each group. (F) Representative trace of voltage responses from the whole-cell patch-clamp showing evoked action potentials from current injection. (G) Inward sodium (Na^+^) and outward potassium (*K*^+^) currents triggered by stepwise depolarization of the cell showing no major difference between the cell lines, *n* = 8 in each group. All data are expressed as mean±the standard deviation.

In order to confirm previous findings that the *α*Syn triplication line does not affect differentiation into DA neurons *in vitro* [35], we performed an experiment with both the AST18 and RC17 lines patterned towards a VM fate using our previously published GMP differentiation protocol [10]. Immunostaining on day 35 showed high expression of neuronal markers TAU and MAP2, as well as the DA marker TH in both cultures (Fig. 1A). Moreover, RC17-derived and AST18- derived cells expressed FOXA2 and GIRK2, indicating subtype-specific maturation (Fig. 1B, C). At this timepoint *α*Syn was expressed in both cultures (Fig. 1D).

We also assessed the functional maturity of the DA neurons at day 35 *in vitro* using whole-cell patch-clamp recordings. This revealed similar resting membrane potential, and both lines displayed the ability to produce evoked action potentials from current injection (Fig. 1E, F). Moreover, cells exhibited voltage gated sodium (Na^+^) and potassium (*K*+) currents with no major differences between the cell lines (Fig. 1G). This demonstrates that both RC17- and AST18-derived cells are capable of maturing into functional neurons under *in vitro* conditions in 35 days.

### *α*Syn triplication VM progenitor cells survive intracerebral transplantation and generate neuron-rich grafts

**Fig. 2:**
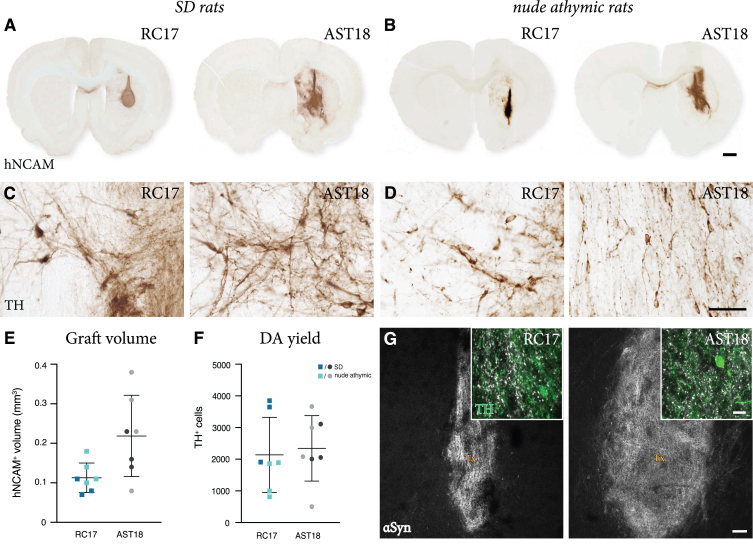
Short term analysis of transplants. hNCAM immunostaining showing surviving grafts (A) in SD rats at 8 weeks and (B) in nude athymic rats at 7 weeks from both RC17 and AST18 VM progenitor cells, scale bar 1 mm. High magnification images of TH immunostaining (C) in SD rats at 8 weeks and (D) in nude athymic rats at 7 weeks, scale bar 50*μ*m. (E) Graft volume quantified showing no major difference between the cell lines in both experiments, *n* = 3 for SD rats (shown in dark blue and dark gray) and *n* = 4 for nude athymic rats (shown in light blue and light gray). (F) Quantification of TH^+^ cells within the graft showing a similar number in grafts from both cell lines in both experiments, *n* = 3 for SD rats (shown in dark blue and dark gray) and *n* = 4 for nude athymic rats (shown in light blue and light gray). (G) Double staining for TH and human *α*Syn showing the distribution of *α*Syn within the transplant (Tx), scale bars 10*μ*m and 100*μ*m. All data are expressed as mean±the standard deviation.

Since AST18 cells have never been investigated after transplantation before, we first tested their capacity to survive and mature after intracerebral grafting. In this experiment, day 16 VM progenitors derived from either RC17 or AST18 were quality controlled according to Nolbrant et al. 2017 (Supplementary Figure 1) and transplanted into the striatum of 6-OHDA-lesioned, immunosuppressed SD rats. The animals in this group were sacrificed at 8 weeks post-transplantation, a timepoint when, based on previous studies, the DA neurons are expected to have formed in the grafts. The grafts were assessed using standard histology. All transplanted rats (3/3 in the RC17 group and 3/3 in the AST18 group) had surviving grafts. The grafts were neuron and DA rich, as evidenced by staining for hNCAM (Fig. 2A) that detects all human neurons, and the DA neuron marker TH (Fig. 2C), thus providing evidence that cells with *α*Syn triplication survive the transplantation procedure and also mature into DA neurons *in vivo*.

To substantiate these findings, we repeated the experiment in 6-OHDA-lesioned nude athymic rats, that do not require daily injections of cyclosporine, therefore allowing for functional studies that require a minimum of 18–20 weeks maturation after transplantation since human cells mature slowly. VM-patterned cells from both lines were transplanted in parallel and a total of 12 rats per cell line were grafted. Four animals in each group were analyzed at 7 weeks post-transplantation to confirm the earlier findings obtained in the immunosuppressed SD rats. All 4 animals in the RC17 group and all 4 animals in the AST18 group had surviving grafts that were neuron and TH rich (Fig. 2B, D). We performed quantification of graft size and TH^+^ neuron content of all rats analysed at 7-8-weeks (*n* = 7 animals per group in total, combining grafts from both SD and nude athymic rats). Although their size and TH^+^ neuron content varied markedly (Fig. 2E, F), as expected in xenografts of this type, the data confirmed that both RC17 and AST18 derived grafts had formed DA neurons with an appearance that is in line with previous reports. We also examined the grafts for *α*Syn and, as expected, we could observe *α*Syn expression in both RC17- and AST18- derived grafts at this time point (Fig. 2G).

These results demonstrate that VM progenitors differentiated from an *α*Syn triplication patient hiPSC line survive and mature into neuron-rich grafts similar to RC17 hESCs after transplantation *in vivo*. This suggests that *α*Syn triplication does not significantly affect the ability of transplanted cells to undergo subtype-specific maturation into DA neurons in the rodent brain.

### VM progenitor grafts derived from an *α*Syn triplication hiPSC line survive long term and are able to mediate functional recovery

**Fig. 3:**
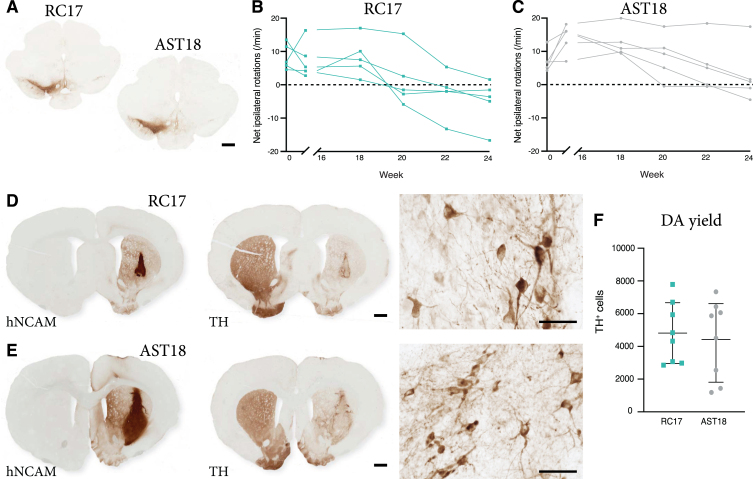
Long term behavioural assessment and analysis of transplants. (A) TH immunostaining showing loss of nigral TH^+^ neurons in the substantia nigra on the lesioned side of the brain, scale bar 1 mm. Net ipsilateral rotation scores at 0, 16, 18, 20, 22, and 24 weeks showing progressive recovery of rotational bias (B) in 5/5 animals in the RC17 group and (C) in 4/5 animals in the AST18 group. (D) hNCAM and TH immunostaining showing surviving grafts at 24 weeks from RC17 VM progenitor cells, scale bar 1 mm. High magnification images of TH immunostaining within the graft at 24 weeks, scale bar 50*μ*m. (E) hNCAM and TH immunostaining showing surviving grafts at 24 weeks from AST18 VM progenitor cells, scale bar 1 mm. High magnification images of TH immunostaining within the graft at 24 weeks, scale bar 50*μ*m. (F) Quantification of TH^+^ cells within the grafts showing no major difference between the cell lines, *n* = 8 in each group. All data are expressed as mean±the standard deviation.

Graft-induced functional recovery was assessed using the amphetamine-induced rotation test in the 6-OHDA lesioned nude athymic rats grafted with RC17-derived and AST18-derived DA progenitors. Animals were tested pre-transplantation (0 weeks) and again at 16, 18, 20, 22, and 24 weeks post-transplantation. Only rats with complete lesions (pre-transplant rotation score >4 net turns/min) and confirmed loss of nigral TH^+^ neurons (*posthoc* histological analysis) (Fig. 3A) were included in this functional analysis, resulting in *n* = 5 in the RC17 group and *n* = 5 in the AST18 group. The rotation data showed that 5/5 of the RC17-derived grafts (Fig. 3B), and 4/5 of the AST18-derived grafts (Fig. 3C), mediated full functional recovery at the endpoint of experiment, i.e., 24 weeks post-transplantation and in a time course that is expected for human DA neurons [41, 42].

Histological analysis was performed on all animals at the end point of the experiment (i.e., also including those with a partial lesion at the start of the experiment). Graft survival was 8/8 in the RC17 group and 8/8 in the AST18 group as assessed by hNCAM and TH staining (Fig. 3D, E). TH immunostaining revealed a more mature neuronal morphology of the DA neurons in both RC17 and AST18 animals (Fig. 3D, E, high magnification) compared to what was observed after 7-8 weeks (Fig. 2C, D), with most mature TH^+^ neurons located at the graft edge (Fig. 3D, E). Quantifications of TH^+^ cells in the grafts showed similar DA content between RC17- and AST18-derived grafts (Fig. 3F). The quantifications also revealed that the one animal in the AST18 group that did not recover in the rotation test had a low number of DA neurons (1440 TH^+^ cells) which is at the threshold for recovery in the rotation test [43].

Thus, both wt hESCs and hiPSCs with an *α*Syn triplication mutation generate functional grafts rich in TH^+^ neurons capable of innervating the surrounding host striatum and mediating functional recovery.

### Grafts derived from *α*Syn triplication VM progenitor cells show evidence of pathological changes

Finally, we performed a detailed investigation of any potential pathology in the AST18- vs the RC17-derived grafts at 24 weeks. For this purpose, grafts were immunolabeled for TH, the microglial marker IBA1, and pSyn (the phosphorylated, pathological form of *α*Syn which is the main component of Lewy bodies).

**Fig. 4:**
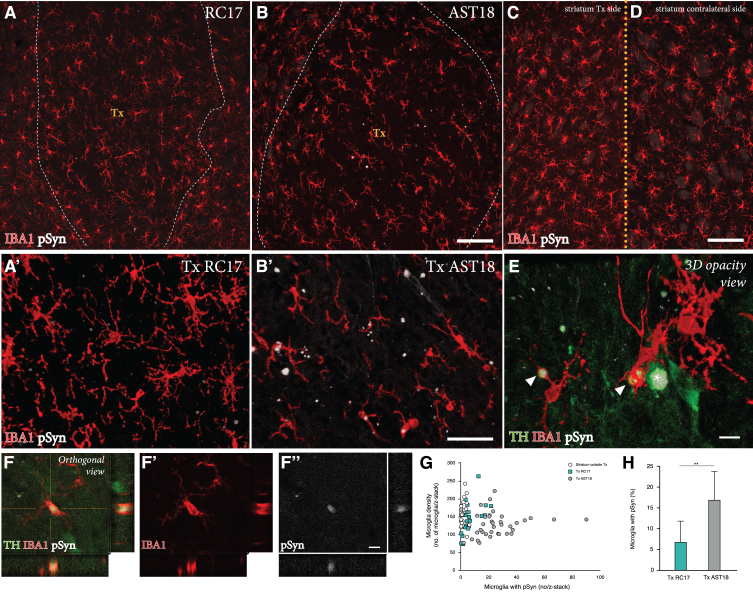
Pathology in microglia 24 weeks after transplantation. IBA1 and pSyn immunostaining within the striatum from (A) RC17- and (B) AST18-derived grafts. The area of the transplant (Tx) is marked with dashed white lines, scale bar 100*μ*m. High magnification images of IBA1 and pSyn double staining within the (A’) RC17 and (B’) AST18 transplant, scale bar 50*μ*m. No pSyn+inclusions were detected in (C) the host striatum outside of the transplant area or (D) on the contralateral side, scale bar 100*μ*m. (E) Arrowheads indicating microglia with pSyn^+^ inclusions, as well as an TH cell containing a pSyn^+^ inclusion marked with an asterisk, scale bar 10*μ*m. (F,F’,F”) Showing a microglial cell containing pSyn^+^ inclusion as evidenced by orthogonal projection, scale bar 10*μ*m. Quantifications showing (G) microglia density plotted against the number of microglia with pSyn^+^ inclusions and (H) the percentage (%) of microglia with pSyn inclusions in RC17- and AST18-derived transplants (Tx) showing a significant difference between the groups (t_ (13) _ = 3.20, *p* = 0.007).

We detected a microglial response to the grafts derived from both cell lines, which is expected with a xenograft transplant (Fig. 4A-D). As illustrated in Fig. 4G, the overall density of IBA1^+^ cells in the grafts was similar to that seen in the surrounding host striatum, and it did not differ between the two graft types. Next, we examined the microglia for expression of pSyn using confocal microscopy. We observed distinct pSyn^+^ inclusions in IBA1^+^ microglia within the AST18-derived grafts (Fig. 4B, B’ and 4E, F), which was markedly different from what we observed in the RC17-derived grafts (Fig. 4A, A’). The frequency of IBA1^+^ microglia with pSyn^+^ inclusions inside the grafts, as assessed in 3D stacks on the confocal microscope (Fig. 4H), showed a significant difference between the two graft types: much fewer in the RC17 grafts as compared to the AST18-derived grafts. No pSyn+inclusions were detected in the host striatum outside of the transplant area (Fig. 4C) or on the contralateral side (Fig. 4D).

**Fig. 5:**
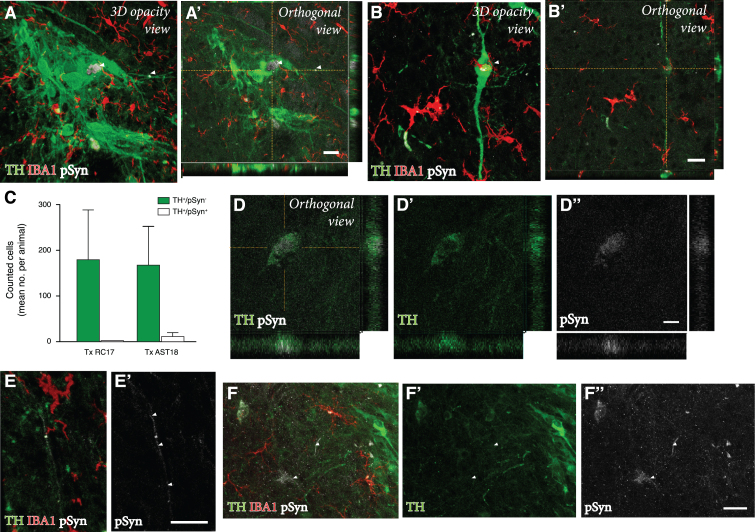
Pathology in DA neurons 24 weeks after transplantation. (A, A’, B, B’) TH, IBA1, and pSyn triple immunostaining within the graft showing TH^+^ neurons containing pSyn^+^ inclusions as evidenced by orthogonal projections, scale bar 20*μ*m. (C) Quantification of the number of pSyn^+^ inclusions in TH^+^ neurons from both RC17 and AST18-derived grafts. TH and pSyn immunostaining showing (D, D’, D”) small granular aggregates of pSyn within a TH^+^ neuron, scale bar 10*μ*m, and (E, E’) a TH-stained neurite containing pSyn^+^ inclusions (indicated by arrowheads), scale bar 20*μ*m. (F) Granular aggregates of pSyn in a weakly stained DA neuron (shown by arrowheads) suggesting down-regulation of TH, scale bar 20*μ*m. All data are expressed as mean±the standard deviation.

To assess any potential disease-related pathology in the DA neurons within the grafts, we analysed the co-expression of TH and pSyn using confocal microscopy. In line with previous transplantation studies of RC17-derived DA transplants in the 6-OHDA model [44], we did not observe any pSyn pathology in TH^+^ neurons in the RC17 group. However, we did find signs of pSyn^+^ inclusions in a small number of the TH^+^ neurons in AST18 grafts (Fig. 5A, B). Quantifications showed that 7% of the TH^+^ neurons (99 out of 1437 TH^+^ neurons counted in 8 different animals) in AST18-derived grafts contained pSyn^+^ inclusions (Fig. 5C). The pSyn^+^ inclusions in the AST18 grafts were most often found within small granular aggregates in the cytoplasm (Fig. 5D) or along neurites (shown by arrowheads in Fig. 5E). We also observed pSyn^+^ inclusions in cells that displayed weak TH^+^ staining (shown by arrowheads in Fig. 5F) indicative of TH down-regulation as part of a degenerative process.

## DISCUSSION

Cell replacement therapy for PD was first explored decades ago, yet a scalable source of cells for transplantation has only recently become available due to advances in stem cell biology. It is now possible to efficiently generate transplantable progenitors, from both hESCs and hiPSCs, that give rise to functional midbrain DA neurons after transplantation into both rodent and primate models of PD [9–11, 28]. This pivotal development has led to the initiation of new clinical trials using allogeneic hiPSCs in Japan (CiRA trial) [45], HLA-matched hESCs in China [46], and others due to be initiated in the next few years in Europe and the United States [47, 48]. In these trials, PD patients will be transplanted with DA progenitors differentiated from either hESCs or hiPSCs (derived from donors without a PD disease background), and patients receiving allogeneic transplants will undergo 1 year of immunosuppression [47].

Personalised stem cell therapy for PD is an attractive future application that is actively under investigation. An autologous grafting strategy in-volves hiPSC generation, differentiation, and transplantation of the patients’ own cells, and would remove the need for immune suppression. To date, one individual with sporadic PD has been reported to have received an autologous cell transplantation, approved by regulatory authorities as compassionate use [16], thereby showing feasibility of the approach. However, given post mortem evidence of slowly developing pSyn pathology in the recipients of fetal VM grafts [17–21], and the observation of disease-associated features appearing when patient-derived hiPSCs are differentiated into DA neurons *in vitro* [22–26], a number of questions relating to the long-term integrity, stability and function of grafts from patient-derived cells remain to be answered.

One key issue to be addressed before initiating clinical trials based on autologous cells are whether patient-derived cells are more prone to develop pathology over extended time periods in the brain, and if they develop pathology in an accelerated manner in an autologous grafting paradigm compared to strategies where cells from healthy donors are used. So far, this has been investigated in very few transplantation studies using cell lines generated from sporadic PD patients. These studies demonstrate that DA progenitors derived from sporadic PD patients are capable of producing DA-rich grafts that mediate functional recovery both in rodent [27–29] and primate models of PD [11]. No obvious signs of pathology have been reported in these studies. However, in a clinical setting the graft should remain healthy and functioning for decades, and this is not possible to predict from these studies. In this study, we therefore used a hiPSC line from a patient with an *α*Syn triplication mutation with the idea that the pathological process is likely accelerated in these cells compared to cells from sporadic PDpatients.

Previous studies have presented conflicting evidence about the DA differentiation efficiency of patient cell lines harboring the *α*Syn triplication mutation. One study reported that *α*Syn triplication impairs neuronal differentiation and maturation *in vitro* [49]. However, other studies have reported that patient cell lines with *α*Syn mutations differentiate with the same efficiency as healthy cell lines regardless of the *SNCA* genotype [22, 26, 35]. Here, we made a comparison of the AST18 cell line (derived from an *α*Syn triplication patient) with that of a clinical grade hESC line with no mutations in known PD related genes. We found no difference in how these cells responded to patterning factors, and both lines generated mature and functional DA neurons *in vitro*, which is in agreement with previous studies [22, 26, 35].

Furthermore, we next showed that *α*Syn triplication cells generate DA-rich grafts also after transplantation into the 6-OHDA preclinical model of PD. At early time points, these grafts behave *on par* with grafts derived from a healthy GMP-grade hESC stem cell line grafted in parallel, and in line with previous transplantation studies using fetal, hESC, and hiPSC-derived grafts [10, 11, 37, 38]. Inaddition, at 24 weeks post-transplantation grafts derived from an *α*Syn triplication patient mediated functional recovery, thereby supporting previous reports that patient-derived DA neurons function in preclinical PD models [27–29].

However, contrary to previous studies using cells derived from sporadic PD patients, we observed the appearance of pathological changes in grafts derived from an *α*Syn triplication patient in the form of pSyn^+^ inclusions present in both reactive microglia and in the cell bodies and/or fibers of TH^+^ neurons indicating that the pathological process is progressing. The dynamics and extent of TH down-regulation vs. DA neuron degeneration is not possible to experimentally assess in our study, but we did observe signs of TH down-regulation in affected cells (see for example Fig. 5F), similar to what has been shown for endogenous DA neurons in AAV-mediated overexpression of *α*Syn *in vivo* [50, 51].

In previous studies *α*Syn pathology has been observed in grafted DA neurons [17–21], but in all these cases the appearance of *α*Syn aggregates reflects transfer of pathology from the *α*Syn overexpressing host brain. In this study, *α*Syn triplication grafts show evidence of pathological changes at 24 weeks post-transplantation despite there being no ongoing pathology in the host brain. The development of *α*Syn pathology in these grafts is most probably due to the 2-fold increase in *α*Syn protein known to be expressed by these cells [26, 35] suggesting that cell intrinsic properties, and increased cellular levels of *α*Syn in particular, can lead to pathological changes within the grafts. Of particular note, is that the limited amount of pathology in the AST18-derived grafts was of no consequence to the function of the graft at this timepoint; however, it cannot be ruled out that the pathology may severely affect the graft and reduce its effectiveness over an extended period of time which cannot be modeled in a xenograft setting. Furthermore, this study was conducted in the 6-OHDA lesion model of PD [52], a toxin based system that induces profound loss of DA neurons accompanied by severe motor deficits, but does not reflect the progressive time course of the disease and lacks pathological hallmarks of the disease process. Therefore, the presence of pathology in the cells may in fact be more aggressive when exposed to the host environment of the PD patient brain, as would be the case in an autologous grafting paradigm. Future studies, therefore, need to investigate patient-derived grafts in more disease relevant models with the presence of proteinopathy and/or inflammation.

Even though this and future studies may indicate that PD patient-derived hiPSCs may not be suitable for immediate use in autologous therapy, there are several ongoing developments in the field that could be used in such cases. For monogenetic cases, gene correction strategies could be employed at the hiPSC stage of cell development [26]. Other examples are drugs or antibodies that prevent Lewy body formation and/or prevent the potential spread of *α*Syn from host to graft that are under development and could be used in combination with autologous grafting in both sporadic and familial patient groups [53, 54]. Future studies could also investigate the effect of engineering *α*Syn null cells for transplantation, as it has been suggested that cells lacking *α*Syn may be resistant to synuclein pathology [26, 55]. These developments could allow for autologous cell therapy and the associated benefits, while minimizing potential pathological effects on the grafted cells and ensuring their long-term function.

## CONFLICT OF INTEREST

MP is the owner of Parmar Cells AB and co-inventor of the U.S. patent application US Patent App. 16/414,848 owned by Biolamina AB, US Patent App. 16/632,229 owned by Miltenyi Biotech and International Application No. PCT/EP2018/062261 owned by New York Stem Cell Foundation.

## Supplementary Material

Supplementary Material
