# Supplementary material for: Grafts Derived from an α-Synuclein Triplication Patient Mediate Functional Recovery but Develop Disease-Associated Pathology in the 6-OHDA Model of Parkinson’s Disease
Source: J Parkinsons Dis. 2021 Apr 13;11(2):515–28. doi: 10.3233/JPD-202366 (PMC8150478; doi:10.3233/JPD-202366)

Supplementary Material

Grafts Derived from an α-Synuclein Triplication Patient Mediate Functional Recovery but Develop Disease-Associated Pathology in the 6-OHDA Model of Parkinson's Disease

Supplementary Figure 1. Analysis of VM progenitor cells at day 16 *in vitro*.
(A) Immunostaining of VM-patterned progenitor cells showing high co-expression of LMX1A/FOXA2 and LMX1A/OTX2. (B) qRT-PCR results of VM-patterned progenitor cells. Gene expression levels are shown as fold change (FC) over undifferentiated stem cell.

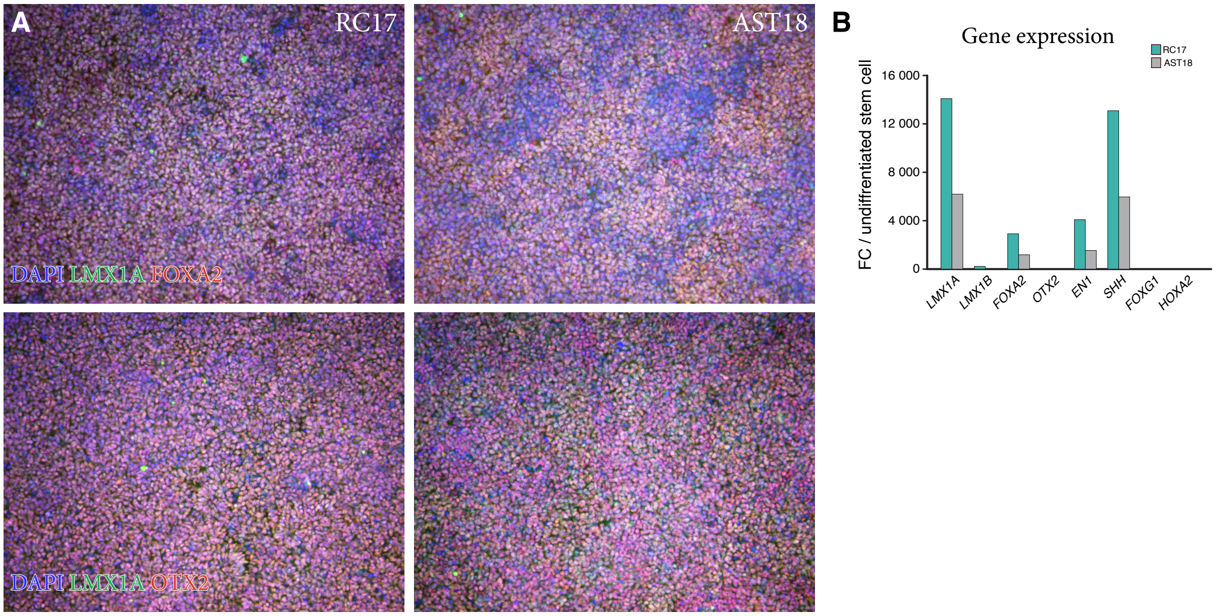

Supplement: Supplementary Material [file jpd-11-jpd202366-s001.pdf]
